# Investigation of the utility of the 1.1B4 cell as a model human beta cell line for study of persistent enteroviral infection

**DOI:** 10.1038/s41598-021-94878-y

**Published:** 2021-08-02

**Authors:** Jessica R. Chaffey, Jay Young, Kaiyven A. Leslie, Katie Partridge, Pouria Akhbari, Shalinee Dhayal, Jessica L. Hill, Kyle C. A. Wedgwood, Edward Burnett, Mark A. Russell, Sarah J. Richardson, Noel G. Morgan

**Affiliations:** 1 Islet Biology Group, Exeter Centre for Excellence in Diabetes (EXCEED), Institute of Biomedical and Clinical Science, University of Exeter Medical School, Exeter, EX2 5DW UK; 2 Culture Collections, National Infection Service, European Collection of Authenticated Cell Cultures, Public Health England (PHE), Salisbury, SP4 0JG UK; 3 Living Systems Institute, University of Exeter, Exeter, UK

**Keywords:** Cell biology, Endocrinology

## Abstract

The generation of a human pancreatic beta cell line which reproduces the responses seen in primary beta cells, but is amenable to propagation in culture, has long been an important goal in diabetes research. This is particularly true for studies focussing on the role of enteroviral infection as a potential cause of beta-cell autoimmunity in type 1 diabetes. In the present work we made use of a clonal beta cell line (1.1B4) available from the European Collection of Authenticated Cell Cultures, which had been generated by the fusion of primary human beta-cells with a pancreatic ductal carcinoma cell, PANC-1. Our goal was to study the factors allowing the development and persistence of a chronic enteroviral infection in human beta-cells. Since PANC-1 cells have been reported to support persistent enteroviral infection, the hybrid 1.1B4 cells appeared to offer an ideal vehicle for our studies. In support of this, infection of the cells with a Coxsackie virus isolated originally from the pancreas of a child with type 1 diabetes, CVB4.E2, at a low multiplicity of infection, resulted in the development of a state of persistent infection. Investigation of the molecular mechanisms suggested that this response was facilitated by a number of unexpected outcomes including an apparent failure of the cells to up-regulate certain anti-viral response gene products in response to interferons. However, more detailed exploration revealed that this lack of response was restricted to molecular targets that were either activated by, or detected with, human-selective reagents. By contrast, and to our surprise, the cells were much more responsive to rodent-selective reagents. Using multiple approaches, we then established that populations of 1.1B4 cells are not homogeneous but that they contain a mixture of rodent and human cells. This was true both of our own cell stocks and those held by the European Collection of Authenticated Cell Cultures. In view of this unexpected finding, we developed a strategy to harvest, isolate and expand single cell clones from the heterogeneous population, which allowed us to establish colonies of 1.1B4 cells that were uniquely human (h1.1.B4). However, extensive analysis of the gene expression profiles, immunoreactive insulin content, regulated secretory pathways and the electrophysiological properties of these cells demonstrated that they did not retain the principal characteristics expected of human beta cells. Our data suggest that stocks of 1.1B4 cells should be evaluated carefully prior to their use as a model human beta-cell since they may not retain the phenotype expected of human beta-cells.

## Introduction

The generation of a human pancreatic beta cell line which reproduces the responses seen in primary beta cells, but is amenable to propagation in culture, has long been an important goal in diabetes research. Access to valid cell models can be expected to extend significantly the scope of studies undertaken to improve the broader understanding of beta-cell biology and a number of groups have established human beta-cell lines for use by the research community^1–8^. In the present work, we chose to utilise one such cell, developed by McCluskey et al^9^, in which primary human beta-cells were electrofused with a pancreatic ductal carcinoma cell, PANC-1, to generate a hybrid named 1.1B4. These cells reportedly retain many of the characteristics of authentic primary beta-cells^10–12^ but they also display the growth characteristics of the parental PANC-1 cell such that they are readily propagated in tissue culture.

Our immediate goal was to test the hypothesis that the process of autoimmunity occurring in people progressing to type 1 diabetes may be triggered by the establishment of an enteroviral infection in their beta-cells. A body of circumstantial evidence supports this hypothesis^13–20^ and much of this has emerged from the detailed study of human pancreas samples recovered post mortem^11–16^. Collectively, however, this work has also prompted a further and much less anticipated conclusion by indicating that the enteroviral infections present in beta-cells may develop in an atypical, persistent, form^15–20^. Verification of this hypothesis would offer new insights into the role of environmental factors in the development of type 1 diabetes but it has been difficult to evaluate fully using human tissue samples because they present the disease process at only a single point in time. The hypothesis is also difficult to test using isolated human islets because primary islet cells do not proliferate readily in culture and there is extensive cell lysis in the outer mantle of islet cells incubated with enteroviruses under in vitro conditions^21^. Therefore, the use of a cultured model human beta cell line offers a potentially important vehicle to progress such studies effectively. Indeed, the 1.1B4 cell line appeared to offer an ideal model system for our purpose^22,23^ since the parental line, PANC-1, has been reported to sustain a persistent enteroviral infection over many months^24^. Accordingly, we sought to use 1.1B4 cells to establish a persistent infection with a strain of enterovirus, Coxsackie B4.E2, which was isolated originally from a child with type 1 diabetes^25^. We were successful in this objective but we also discovered a number of features of the cellular response which were inconsistent with those expected of human beta cells.

Given the unexpected nature of our results, we were prompted to investigate the characteristics of 1.1B4 cells more thoroughly and were led to the conclusion that stocks of these cells are composed of heterogeneous populations of cells arising from both humans and rodents. Indeed, it became clear that simple propagation of the cells over successive passages can lead to a diminution in the sub-population of the human cells, leading to an expansion of the proportion of their rodent counterparts. These findings were confirmed by multiple methods and in a number of cellular stocks, including those held by the European Collection of Authenticated Cell Cultures (ECACC).

## Results

### Cultured 1.1B4 cells can sustain a persistent enteroviral infection over long periods

**Figure 1:**
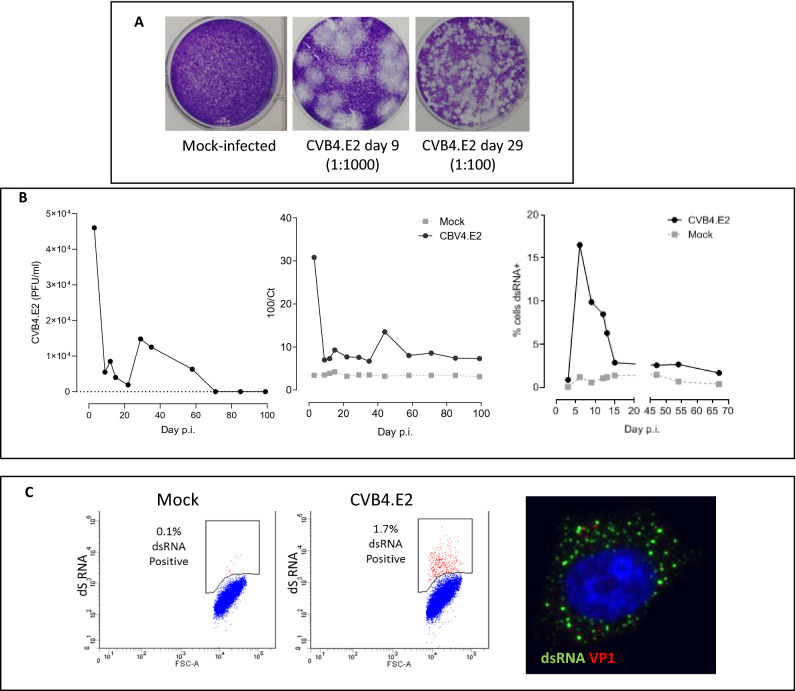
Progression of enteroviral infection in 1.1B4 cells exposed to Coxsackie B4.E2. (**A**) Cell culture medium collected from 1.1B4 cells was utilised in virus infectivity assays with HEp-2 cells. Viral plaques were examined after plating of culture medium (diluted as shown) that had been collected from mock infected cells (left panel) or from cells on either day 9 (middle plate) or day 29 (right plate) post-infection and incubated on lawns of HEp-2 cells. Representative results from an individual experiment are presented. (**B**) One Step qRT-PCR was performed with CBV4-specific primers using RNA harvested from culture medium of CVB4.E2 or mock infected 1.1B4 cells. Representative results from an individual experimental run are shown and are presented as relative Ct values (middle panel) in comparison with quantified plaque forming units of virus (left panel). The percentage of dsRNA positive (virally-infected) cells at each time point is shown in the right panel. (**C**) A representative example of CBV4.E2 or mock infected cells stained using an antibody raised against dsRNA and assessed by flow cytometry at day 64 post infection. The right panel illustrates cells immunostained to detect VP1 and dsRNA (imaged on day 12 post infection).

As a means to study the ability of 1.1B4 cells to sustain and propagate a productive enteroviral infection, the cells were exposed to a strain of Coxsackie B4 (CVB4.E2) at an MOI of 0.01 and the progress of the infection followed over time. This was achieved in a number of ways including by the amplification of viral RNA present in the cell culture medium (using PCR) and by the measurement of infective virus (using plaque assays) harvested from supernatants collected sequentially on multiple days beyond the initial infection. In the early phases of infection (days 1–10) high viral titres were detected by PCR and by plaque assay (Fig. 1A,B). However, the viral titres declined and viral plaque sizes become progressively smaller over time. Ultimately, by day 60, no visible plaques were generated (Fig. 1) upon harvesting of the culture medium and subsequent plating on a lawn of HEp-2 cells, although viral RNA could still be measured in such samples, by PCR (Fig. 1B). These data suggest that the initial acute infection had resulted in the early generation of large numbers of viable viral progeny but that this process was not sustained. Rather, the virus had apparently established a persistent mode of infection (with limited generation of infective progeny) following the initial acute phase. In support of this, flow cytometric analysis of cells harvested at increasing duration of culture revealed that the viral product, dsRNA, (detected using antibody J2) was present at very high levels during days 1–10 post infection but declined thereafter (Fig. 1B,C). Importantly, however, it was never lost completely from the cultures and could be detected in a small proportion of the cells (1–2%) at all stages of the experiment, including as late as day 64; well beyond the period when productive virus was produced (Fig. 1C). The generation of viral capsid protein VP1 could be detected in parallel with dsRNA in some samples (Fig. 1C).

### Expression of interferon stimulated genes in 1.1B4 cells during the development of a persistent enteroviral infection

**Figure 2:**
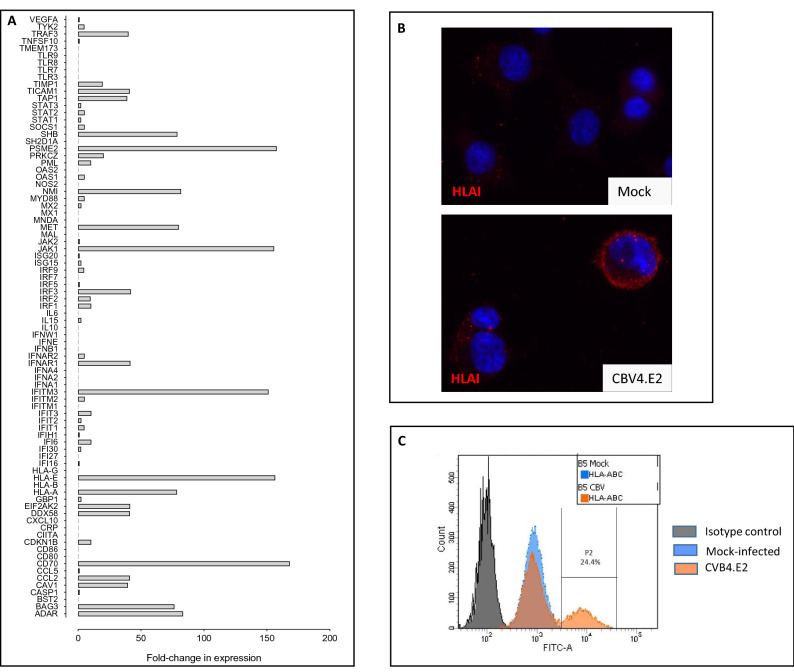
Induction of viral response genes following infection of 1.1B4 cells with Coxsackie B4.E2. (**A**) A human RT2-Profiler Type 1 IFN PCR array was used to monitor the expression of target viral response genes in mock or CBV4.E2 infected 1.1B4 cells harvested on day 44 post infection. (**B**) HLA Class I expression was studied by immunocytochemistry in mock or CVB4.E2-infected 1.1B4 cells harvested on day 71 post-infection. (**C**) Flow cytometry was used to measure cell surface expression of HLA class I on mock (blue) and CVB4.E2 infected (orange) 1.1B4 cells harvested on day 98 post infection. The profile of cells labelled with isotype control antiserum is shown in grey.

To establish whether the development of a persistent enteroviral infection was associated with the induction of interferon responses in 1.1B4 cells, an RT-Profiler Type 1 interferon array was used. This allowed a comparison of the expression of interferon stimulated genes between mock infected and persistently infected 1.1B4 cells at day 44 post infection. Analysis of gene expression revealed an apparent upregulation of several interferon-stimulated genes in the persistently infected cells (Fig. 2A) including HLA-A, a well-recognised interferon-stimulated gene in beta-cells (Fig. 2A,B). Intriguingly, however, the use of immunofluorescence staining and flow cytometry revealed that, while HLA-A was increased on the surface of certain cells in the culture, it was not abundant on all cells (Fig. 2B,C).

**Figure 3:**
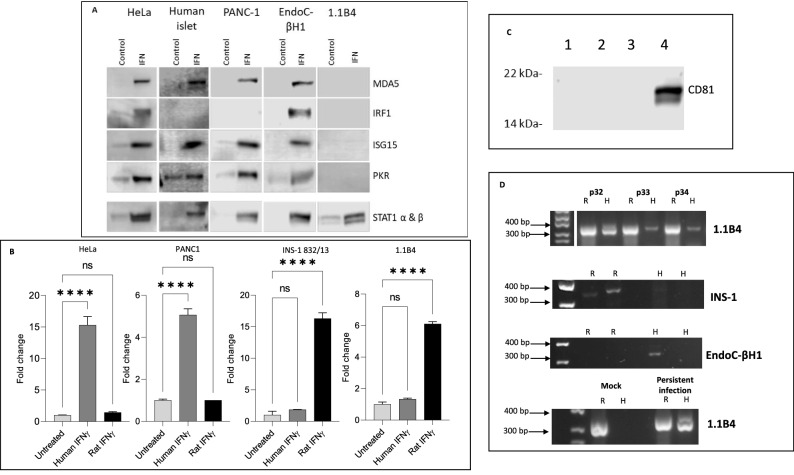
Species specificity of anti-viral responses measured in 1.1B4 cells by comparison with those seen in a range of additional cell types. (**A**) The induction of a selection of viral response proteins was studied in 1.1B4 cells following exposure to human interferon-alpha (IFN; 1000U/ml) for 24 h. The profile was compared with that seen in EndoC-βH1, PANC-1 and HeLa cells as well as in isolated human islets, incubated under the same conditions. Controls received vehicle alone. Images of full blots are available in supplementary information. (**B**) 1.1B4, PANC-1, HeLa or INS-1 832/13 cells were each transfected with a luciferase reporter construct under the control of a GAS promoter and then incubated with either 20 ng/ml human IFNγ or rodent IFNγ for 24 h. Reporter activity was measured in cell extracts and expressed relative to that measured in untreated cells. **** = *P* < 0.0001; ns = not significant. (**C**) Exosomes were harvested from the culture medium of 1.1B4 cells (lanes 1–3) or from PANC-1 cells (lane 4) and protein extracts probed for the presence of the marker protein CD81 using a human-specific antiserum. Immunoreactive proteins were detected by Western blotting. (**D**) DNA was harvested from 1.1B4 cells at increasing passage, as shown (upper panel) and amplified using primers targeting vomeronasal receptor sequences of either rat (R) or human (H) origin. INS-1 832/13 (second panel) and EndoC-βH1 cells (third panel) were used as rat or human positive controls, respectively. Either mock or CVB4.E2-infected 1.1B4 cells were also harvested and analysed at 71 days post infection (lower panel). Images of the whole gel are available in supplementary information.

In an attempt to understand more completely the propensity of 1.1B4 cells to sustain a persistent enteroviral infection, the induction of interferon stimulated gene products (ISGs) was studied at the protein level. The expression of relevant gene products was compared across a range of different human cells lines (PANC-1, EndoC-βH1 and HeLa) and with isolated human islets incubated under culture conditions, following addition of IFNα (Fig. 3A). Upon exposure to human IFNα for 24 h, the expression of MDA5 (encoded by *IFIH1*), IRF1, ISG15, PKR (encoded by *EIF2AK2*), and STAT1 was assessed by Western blotting. As expected, the majority of the cell lines (as well as human islets) responded to IFNα by marked upregulation of these gene products (Fig. 3A). However, to our surprise, 1.1B4 cells appeared resistant to IFNα in that, although they demonstrated an increase in STAT1, none of the other proteins tested were detected.

To investigate this conundrum more thoroughly, we performed additional studies with HeLa and PANC-1 cells in parallel with a rodent beta-cell line, INS-1 832/13. Each was treated with human or rodent interferon-gamma (according to their species of origin) and the activity of a reporter construct driven by the binding of STAT1 homodimers (firefly luciferase under the control of a Gamma Activated Sequence (GAS) promoter) was measured. As expected, HeLa and PANC-1 cells responded to human IFNγ with a marked increase in luciferase reporter activity (Fig. 3B). By contrast, neither of these cells responded to rodent IFNγ, suggesting that there is extreme species specificity at the level of the IFNγ receptor (Fig. 3B). In support of this conclusion, luciferase reporter activity was enhanced markedly when INS-1 832/13 were exposed to rat IFNγ, whereas they failed to respond to human IFNγ (Fig. 3B). A major surprise emerged when equivalent experiments were then performed in 1.1B4 cells. These displayed only a minimal response to human IFNγ whereas the luciferase activity was increased markedly when the cells were treated with rat IFNγ (Fig. 3B).

In considering the potential implications of these data, we next explored the expression of a range of proteins in 1.1B4 cells that could be detected in a species-specific manner using highly selective antisera in Western blotting studies. Focusing on CD9, CD81, CD63 and HSP90 as representative molecules expected to be present in exosomes exported from beta-cells^26,27^, we harvested the exosomal fraction (vesicle size: 50–130 nm) from the culture medium and established that none of the target proteins was recognised by human-specific antisera when 1.1B4 cells were used. By contrast, each of these proteins was detected when PANC-1 cells were employed as the source of exosomes. A Western blot probed for human CD81 is shown as representative of these data in Fig. 3C.

### Amplification of species-specific vomeronasal receptor nucleic acid sequences from cultured cells

Given the modest proportion of cells expressing elevated HLA-I after viral infection, the paucity of response to either human IFNα (monitored by Western blotting) or human IFNγ (using reporter assays) and the lack of immunoreactivity of human proteins exported from 1.1B4 cells in exosomes, we were drawn towards the possibility that the cultures may have become contaminated and that human cells were present in the minority. To assess this, we employed a species-specific PCR assay targeting the transcripts encoding vomeronasal receptors. This approach was selected because the assay had been validated in earlier work as a sensitive means to probe for species contamination in cell culture systems^28^. Accordingly, genomic DNA was extracted from 1.1B4 cells passaged for different periods and employed as template to amplify target vomeronasal receptor sequences using rat or human-specific primers. Importantly, when using DNA extracted from cells at the earliest passages of 1.1B4 cells tested, both human and rodent vomeronasal receptor sequences were amplified (Fig. 3D). By contrast, when RNA extracted from EndoC-βH1, PANC-1 (not shown) or INS-1 cells was tested, only a single amplicon was generated and this corresponded precisely with the expected species in each case (Fig. 3D). Further analysis of 1.1B4 cells grown over successive passages revealed a rapid loss of the band corresponding to the human vomeronasal receptor sequence while the equivalent rat sequence was retained (Fig. 3D). This suggested the unexpected conclusion that the 1.1B4 cells available to us, are not a pure preparation of human cells but, rather, they contain populations of cells that are of either human or rodent origin.

Having made these observations, we then re-visited the cells used in our initial enteroviral infection studies and analysed the vomeronasal receptor profiles in mock- and persistently infected 1.1B4 cells. By so doing, it was established that those cells which had been mock transfected and then maintained in culture for more than 68 passages were exclusively rodent in origin (Fig. 3D). By contrast, those cultures which had been established in parallel but then infected with enterovirus, retained detectable human vomeronasal receptor sequences, together with equivalent rat sequences (Fig. 3D) implying the presence of cells from both species. Thus, the 1.1B4 cultures which had sustained a persistent enteroviral infection appeared to have also retained a proportion of human cells, despite the extended culture period.

Having reached these disconcerting conclusions, we reported our findings to the European Collection of Authenticated Cell Cultures, from whom our original stocks of 1.1B4 cells had been obtained. They undertook an independent analysis of separate stocks held in their collection using short tandem repeat (STR) analysis. Their analysis confirmed that the original culture stocks of 1.1B4 cells held in their biobank contain both rat and human DNA. Together the results imply that the human 1.1B4 cell stocks generated originally by electrofusion of two human cells, had become contaminated with an unidentified rodent cell at an early point in the process of propagation and expansion.

### Cloning and characterisation of human 1.1B4 sub-populations following single cell sorting

**Figure 4:**
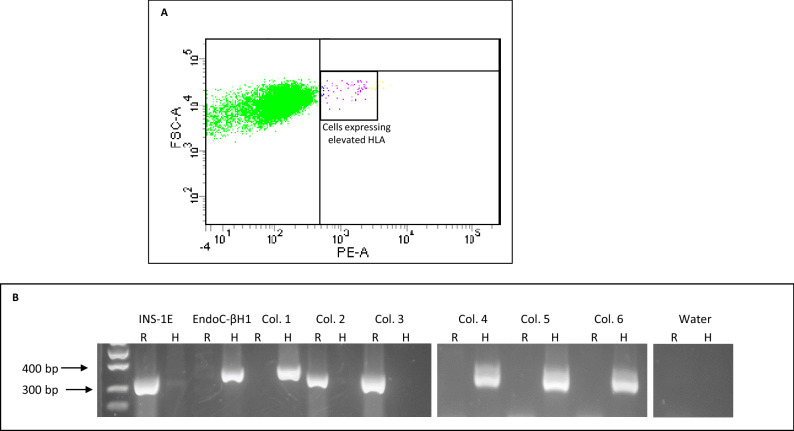
Sorting and expansion of 1.1B4 cell clones expressing human HLA class I. (**A**) 1.1B4 cells (passage 28) were treated with 1000 U/ml human interferon-alpha for 24 h then sorted by flow cytometry after staining with a human-specific anti-HLA class I antibody. Cells which had upregulated HLA class I (purple and yellow symbols) were selected and seeded into single cells in 96 well plates and expanded. (**B**) After expansion, 6 colonies were selected and genomic DNA isolated for analysis by PCR using species-specific (R – rat; H- human) vomeronasal receptor primers. INS-1E (rat) and EndoC-βH1 (human) cells were analysed in parallel as controls. Images of the whole gel are available in supplementary information.

Based on these unexpected discoveries, and the apparent utility of the 1.1B4 cell line for study of persistent enteroviral infection, we felt it worthwhile to employ a single cell cloning strategy in an attempt to re-establish a pure population of human cells. Accordingly, early passage (p28) 1.1B4 cells were treated with human IFNα for 24 h and then analysed by flow cytometry to detect the expression of HLA-class I molecules on the cell surface, using a selective human anti-HLA-I antibody (Fig. 4A). Using this methodology, we found that ~ 1% of the 1.1B4 cells upregulated their expression of HLA-I in response to human IFNα. Using a flow cytometer, these cells were then selected and sorted directly into the individual wells of 96 well plates (one cell per well). From two such plates, six colonies were expanded successfully and their genomic DNA extracted for analysis using species-specific primers amplifying vomeronasal receptors. Among these six colonies, two (colonies 2 and 3) were shown to contain only rat DNA while the remaining four were human (Fig. 4B). Two of the human colonies were expanded further and then transported to ECACC for independent validation using STR analysis. This confirmed their origin as exclusively human.

**Figure 5:**
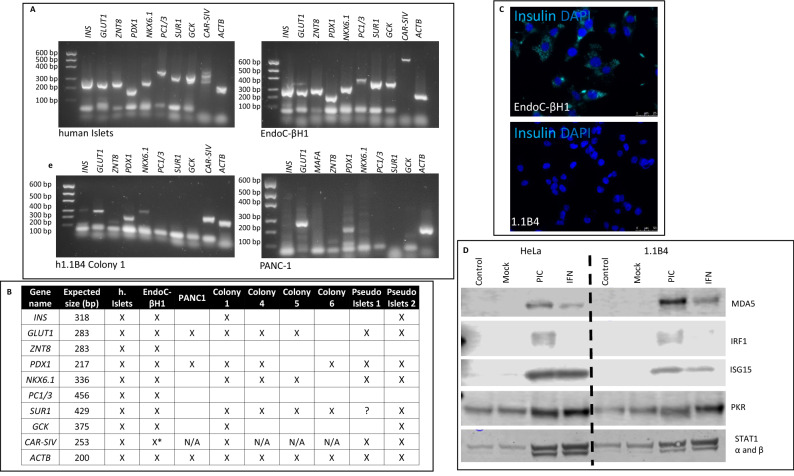
Gene expression profiles of expanded human 1.1B4 cell clones. (**A**) mRNA was extracted from isolated human islets, EndoC-βH1 cells, human 1.1B4 cells (data for colony 1 are shown as a representative example) and PANC1 cells. The expression of a selection of genes frequently expressed in β-cells was assessed by RT-PCR. Images of the whole gels are available in supplementary information. (**B**). Summary graphic showing the expression of a selection of β-cell genes expressed in isolated human islets, EndoC-βH1 cells, PANC-1 cells or in each of 4 colonies of expanded h1.1B4 cells (colonies 1, 4, 5, 6) configured either in monolayers or (in the case of colony 1) as pseudoislets. (**C**) Insulin expression (light blue) was investigated in EndoC-βH1 and h1.1B4 cells (colony 1) by immunocytochemistry. Nuclei were stained with DAPI (dark blue). (**D**) HeLa and h1.1B4 cells (colony 1) were treated with vehicle (control) or with interferon-alpha (IFN; 1000 U/ml) for 24 h and lysed. In parallel, additional cells were either mock transfected or transfected with polyI:C (PIC; 0.1 mg / ml) and incubated for 24 h. Western blotting was employed to monitor the expression of MDA5, IRF1, ISG15, PKR and STAT1. Images of the whole blots are available in supplementary information.

It then became important to establish whether the cloned and expanded human 1.1B4 (“h1.1B4”) cells retained the profile of beta-cell characteristics reported following their initial derivation^9^. To address this, RT-PCR amplification was performed to detect a range of key β-cell genes. As positive controls, RNA extracted from samples of isolated human islets and EndoC-βH1 cells were analysed in parallel (Fig. 5A). A parental PANC-1 cell line, equivalent to that used in the initial electrofusion protocols, was also included as a further control. This qualitative analysis revealed that, while human islets and EndoC-βH1 cells expressed a full complement of the beta-cell genes examined (including *INS, GLUT1, ZNT8, PDX1, NKX6.1, PC1/3, SUR1, GCK, CAR-SIV, ACTB*) the majority of these were absent from or, at best, only weakly expressed in h1.1B4 cells (Fig. 5A,B). Upon comparison of the various gene transcripts amplified among the cell types studied, it was evident that h1.1B4 cells were most similar to PANC-1 cells in terms of their expression profiles (Fig. 5B).

To verify these conclusions and to establish more directly whether the newly derived clones of h1.1B4 cells express insulin at the protein level, immunocytochemical studies were undertaken. For these experiments, a single clone of h1.1B4 cells was selected based on its profile of beta-cell gene transcripts (Fig. 5A,B) and this was studied in parallel with EndoC-βH1 cells. Immunostaining of each cell type revealed that, among EndoC-βH1 cells, the majority contained abundant levels of immunoreactive insulin. By contrast, h1.1B4 cells were devoid of insulin immunoreactivity (Fig. 5C). We also examined whether h1.1B4 cells were responsive to IFNα by monitoring the expression of various downstream interferon-response proteins (Fig. 5D). This confirmed that, whereas the unsorted (mainly rodent) 1.1B4 cells had been largely unresponsive to human IFNα (Fig. 3) the cloned h1.1.B4 cells showed more robust responses with clear increases in the production of human MDA5, ISG15, PKR and STAT1 (Fig. 5D). The cells also responded to transfection with polyI:C (Fig. 5D).

Although it was clear that our cloned h1.1B4 cells do not produce measurable amounts of immunoreactive insulin, we were still keen to explore whether they might retain an intact stimulus-secretion coupling pathway, despite their deficit in insulin biosynthesis. To examine this, we made use of a construct encoding human growth hormone (hGH) and transfected this into h1.1B4 cells prior to stimulation and assessed their secretory function by measurement of hGH release (by ELISA).

**Figure 6:**
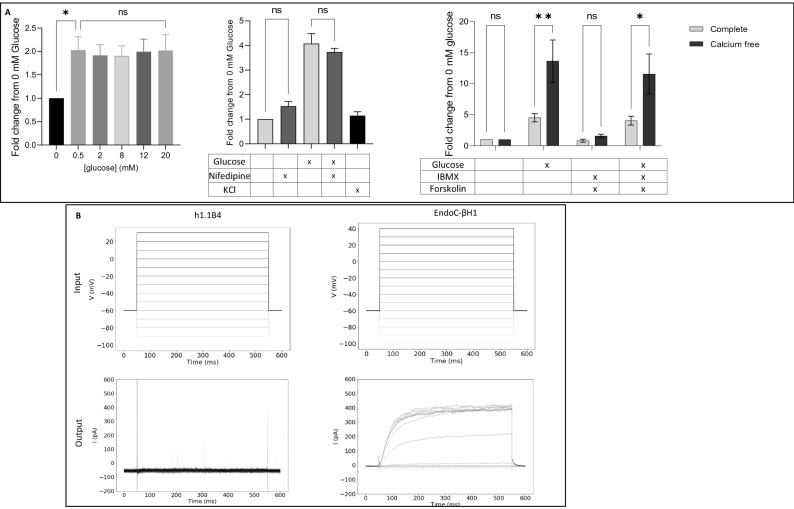
Secretory and electrophysiological characteristics of human 1.1B4 cells. (**A**) h1.1B4 cells (colony 1) were transfected with a plasmid encoding hGH for 48 h prior to incubation with increasing glucose concentrations (left panel); 0 or 20 mM glucose in the absence or presence of either nifedipine (1 μM) or 30 mM KCl (middle panel); 0 or 20 mM glucose in the absence or presence of 1 mM CaCl_2_, 10 μM forskolin or 100 μM 3-isobutyl 1-methyl xanthine (IBMX; right panel). *P* < 0.05, **P ≤ 0.005; ns = not significant. (**B**) Whole cell patch clamp analysis was performed on h1.1B4 cells (colony 1) and EndoC-βH1 cells and the current flow recorded following the application of voltage steps. Input voltage is shown in the upper panels and resultant current flow in the lower panels. Data are from a single experiment representative of at least 9 individual cells in each case.

Initially, the cells were exposed to increasing glucose concentrations (Fig. 6A) and, although a basal release of hGH was measured from transfected cells when the glucose concentration was raised from zero to 0.5 mM, no further increase in hGH secretion was detected during acute stimulation experiments, across the range 0.5–20 mM glucose. The cells were similarly unresponsive to a depolarising concentration of KCl (30 mM) and it was found that the rate of secretion was not attenuated when cells were treated with the L-type Ca^2+^ channel blocker, nifedipine, nor when they were incubated in calcium depleted medium (Fig. 6A). Agents mediating a rise in intracellular cAMP (forskolin, IBMX) were also unable to promote a significant increase in hGH secretion from h1.1B4 cells (Fig. 6A). Surprisingly, the rate of hGH secretion measured in the presence of 20 mM glucose, IBMX and forskolin was enhanced under Ca^2+^-depleted conditions. Finally, studies were undertaken to explore the electrophysiological properties of this clone of h1.1B4 cells, using whole patch clamp techniques (Fig. 6B). Despite the application of increasingly depolarising voltages, no changes in intracellular currents were recorded. By contrast, EndoC-βH1 cells responded with evidence of marked current flow (Fig. 6B).

## Discussion

The extremely limited availability of human pancreatic beta-cell lines amenable to manipulation in vitro presents a clear barrier to progress in diabetes research. Primary human islets represent a gold standard for the study of beta-cell function^29^ but these are available only intermittently. Moreover, even when they can be made available following islet isolation from organ donors, primary beta-cells are not well suited to long term studies conducted in vitro because they display little propensity to undergo mitosis^29^. This is a particular issue when developing model systems to study persistent viral infection since it is desirable to establish and maintain such infections in cells that are dividing and which can be readily manipulated genetically. Very few candidate human beta cell lines exist although the emerging family of EndoC cells developed by Scharfmann and colleagues, offer great potential^6,7,30^. However, in our hands, the most widely used of these cells (EndoC-βH1) appear exquisitely sensitive to the detrimental effects of enteroviral infection such that the majority are killed during the acute phase of infection, even when very low MOIs are employed.

By contrast, clonal lines of pancreatic ductal carcinoma cells, such as PANC-1, are more robust and have been shown by others to sustain a persistent enteroviral infection over many months^24^. Therefore, the advent of a hybrid cell line obtained by fusion of PANC-1 with a primary human beta cell and which retains certain critical features of both, seemed to offer a unique opportunity to study the development of persistent beta-cell enteroviral infection in a physiologically relevant model. The 1.1B4 cell line represents exactly such a cell since it has been reported to synthesise and secrete insulin in a glucose-responsive manner while retaining the growth characteristics of the PANC-1 cell^9,12^.

In view of this promise, we obtained a supply of 1.1B4 cells with the intention to harness their utility to study the response of beta-cells to enteroviral infection in vitro. Initial studies were promising in that the cells were amenable to infection with a diabetogenic enterovirus, CVB4.E2^25^. In addition, we were able to establish conditions under which an initial infection protocol employing a low MOI, allowed the surviving cells to transition effectively between the early acute phase of infection (during which there was significant viral replication and extensive cell loss) to a more persistent phase which could be sustained over many weeks. As such, this appeared to provide a useful model in which to study the molecular basis of the persistence mechanism in a manner which is relevant to that which may occur in vivo in the beta-cells of people progressing to type 1 diabetes^19,20,22,23^.

Unfortunately, our initially optimistic view was subsequently tempered by a number of unexpected findings; not least that the cells^31^ available to us were primarily rodent in origin with very few human cells present in the cultures. Our data are consistent with two possibilities: either that the cells had been hybridised to contain both rodent and human DNA or that the cultures had become contaminated with cells of each species. To distinguish between these possibilities, we initially sorted the cells using flow cytometry and grew up new colonies from each of a large number of single cells. In all cases examined, the resultant clones contained only rodent DNA effectively eliminating the possibility that the original cells may represent human-rodent hybrids. As a result, we concluded that the 1.1B4 cell stocks used in our studies almost certainly comprise distinct populations of both rodent and human cells and we reported these findings to the European Collection of Authenticated Cell Cultures (ECACC) from whom our original stocks were obtained. They then employed STR analysis to confirm that multiple stocks held in their archive collection represent mixtures containing both human and rodent DNA sequences, consistent with the presence of at least two populations of cells. As a result, they have withdrawn 1.1B4 cells from their supply catalogue and have recommended that a range of new measures be introduced to verify the authenticity of hybrid cell lines held in culture collections^32^.

Having reached these unexpected conclusions, we were still intrigued to note that we had succeeded in establishing a persistent infection in cultured 1.1B4 cell clones using a human Coxsackie virus, CVB4.E2. This, in turn, suggested the possibility that the persistence may have been sustained by the small population of human cells present within the cultures rather than by the bulk population of rodent cells. In support of this notion, we found that mock infected cultures did not retain human DNA sequences whereas the corresponding cultures which had been transduced with enterovirus, retained human DNA sequences over many weeks. Thus, it appeared that the human cells were lost (presumably by competition) from mock infected cultures but retained in virally infected cultures. As such, we were drawn to the possibility that human beta cells might gain an undefined selective advantage during the establishment of persistent enteroviral infection and that authentic 1.1B4 cells (human in origin) might still represent a very informative experimental model. Therefore, we sought to clone out human cells from a low passage culture stock of 1.1B4 cells using flow cytometry.

It was then important to characterise the newly derived human cell populations to establish the extent to which they retain beta-cell characteristics and might prove useful for study as a quasi-physiological model system. Unfortunately, we discovered that the cells expressed only minimal amounts of insulin mRNA, they did not contain immunoreactive insulin and their gene expression profile bore little resemblance to that expected of an authentic human beta-cell. Despite this, we pursued studies to establish whether they might retain a functional pathway for regulated secretion by taking advantage of the opportunity to transfect the cells with a human growth hormone construct whose secretion could be evaluated using ELISA^33,34^. The cells were readily transfectable and release of hGH could be detected but the rate of hormone secretion was not influenced by variation in the glucose concentration present in the culture medium between 0.5 and 20 mM or by manoeuvres designed to regulate the rate of Ca influx. Direct analysis of the electrophysiological properties of the cells using patch-clamp methodologies also failed to reveal any of the voltage-regulated membrane currents normally characteristic of beta-cells^4,35–39^. Thus, we were forced to the disappointing conclusion that, even after selective cloning of the sub-population of human cells present in one of the original stocks of 1.1B4 cells held within ECACC, the resultant clonal lines retain very few, if any, of the features expected of human beta-cells.

These are important outcomes since they emphasise that extreme caution should be exercised when interpreting data obtained on the beta-cell characteristics of 1.1B4 cells. In drawing this conclusion, we do not intend to imply that all stocks of these cells are necessarily contaminated with non-human cells and we cannot be certain how or where this contamination occurred. Importantly, we do not suggest that the original report describing the production of human 1.1B4 cells^9^ is called into question. Nevertheless, the fact that all stocks examined by ECACC contained rodent DNA sequences implies that the contamination occurred as an early event during propagation of the cells. On the basis of our experience, we urge that any 1.1B4 cells intended for use as an experimental human beta-cell model should be characterised fully and extensively by the recipient laboratory prior to their use.

## Methods

### Cell culture

1.1B4 cells were obtained from ECACC. HeLa and PANC-1 cells were from the American Tissue Culture Collection (ATCC CCL-2) and ECACC (#87092802), respectively while EndoC-βH1 cells were provided by Prof R Scharfmann via Univercell-Biosolutions (Toulouse, France). INS-1 832/13 cells were a kind gift from Prof Chris Newgard (Duke University, Durham NC). HEp-2 cells were obtained from the American Tissue Culture Collection (ATCC CCL-23) and isolated human islets were kindly provided from the JDRF-funded UK islet isolation programme by Prof Paul Johnson & Dr Steve Hughes (Oxford University, UK).

1.1B4 and INS-1 832/13 cells were cultured in RPMI 1640 media containing 11.1 mmol/L D-Glucose (Gibco), supplemented with 100U/mL Penicillin and 100 µg/mL Streptomycin, 2 mmol/L l-glutamine and 10% heat inactivated foetal bovine serum (FBS). The medium used to grow INS-1 832/13 cells was further supplemented with 50 µmol/L β-mercaptoethanol, 10 mmol/L Hepes and 1 mmol/L Sodium Pyruvate. HeLa and PANC-1 cells were cultured in DMEM medium supplemented with 100 U/mL Penicillin and 100 µg/mL Streptomycin, 2 mmol/L l-Glutamine and 10% FBS. EndoC-βH1 cells were cultured as described by Ravassard et al. (2011)^30^ using DMEM medium (Gibco, UK) containing 5.5 mmol/L D-glucose, 100U/mL Penicillin, 100 µg/mL Streptomycin, 2% BSA (fraction V, fatty acid free), 50 µmol/L β-mercaptoethanol, 5.5 µg/ml Transferrin, 6.7 ng/mL Sodium Selenite and 10 mmol/L Nicotinamide. HEp-2 cells were grown in DMEM medium containing 10% heat inactivated FBS, 1% l-glutamine and antibiotics.

### Propagation of enterovirus stocks

HEp-2 cells were infected with Coxsackie virus strain CBV4.E2 (kindly provided by Prof. Didier Hober, Lille University, France). The cells were incubated for 3–4 days until most had detached from the substrate. The culture medium was not changed during this time. Following this period, the cultures were frozen and thawed 3 times then centrifuged (1000×*g*; 10 min) to clear the lysate prior to aliquots being stored at − 80 °C. The viral titre was estimated using a standard plaque assay.

### Infection in 1.1B4 cells with CVB4.E2

1.1B4 cells were seeded at 60% confluency then incubated for 18–24 h prior to infection with CVB4.E2, diluted in RPMI-1640, to achieve an MOI of 0.01. Mock infected cells received vehicle alone. The mock and infected cell lines were maintained and passaged independently but in parallel. 24 h post infection, the cells were washed extensively in cold PBS and fresh medium added. Cells were maintained until day 3 post infection when initial samples were collected. Cells to be propagated further were scraped from the substrate, gently re-suspended in fresh medium then seeded into culture flasks at 15–25% confluency (mock infected) or 40–70% confluency (CVB infected cells) and cultured as appropriate.

### Isolation and quantification of viral RNA

Cell culture medium was harvested and centrifuged (1000*g* for 5 min) then stored at − 80 °C. RNA was isolated using the PureLink Viral RNA Mini Kit procedure (Invitrogen) according to the manufacturer’s instructions. Cell lysates were prepared in RLT buffer, stored at − 80 °C and subsequently processed using the RNeasy Mini Kit (Qiagen) protocol. RNA was quantified by RT-PCR using the RNA Ultrasense One-Step System (Invitrogen) using half the reaction volumes specified in the standard protocol. Virus specific primers were used, forward gctgaaggagaaaccgttcgtta, reverse aacctagtaacaccatgaacgttgc and probe cccggctaactacttcg.

### Immunofluorescence staining of fixed cells

1.1B4 cells were grown on round coverslips in 24 well plates. Cells were fixed with 4% paraformaldehyde on ice for 20 min and stored at 4 °C under PBS until stained. Cells were stained overnight at room temperature using a dsRNA specific antibody, J2 (SCICONS, Szirák, Hungary) at a dilution of 1:1000. Immunostaining was detected with goat anti-mouse Alexa488 at 1:400 dilution for 1 h at room temperature. For dual staining, anti-dsRNA (J2) was combined with anti-VP1 antiserum. Insulin was detected with Agilent A0564 (1:360) and goat anti-guinea-pig Alexa647 (1:400). DNA was stained with DAPI. All antibodies were diluted in 10% donor calf serum, 0.1 M lysine and 0.2% Triton in PBS.

### Quantification of infectious virus

Samples of media were collected from virally and mock-infected cells, centrifuged (1000*g* for 5 min) and stored at − 80 °C. A standard plaque assay was used in which HEp-2 cells were grown to 95% confluency in six well plates and incubated with 1 ml of harvested medium for 4 h at 37 °C. The medium was removed and wells overlaid with 1% agarose in EMEM containing 20% FBS and 4 mM glutamine. Plates were incubated at 37 °C for 4 days prior to fixing with 10% PFA (1 ml per well) at room temperature for 2 h. The agarose overlay was removed and cells were stained with 1% crystal violet for 30 min.

### Flow cytometry

1.1B4 cells were treated for 24 h with 1000 U/ml human IFNα. Cells were harvested with trypsin, centrifuged (200*g* for 5 min) then washed and re-suspended in FACS buffer. They were subdivided and incubated with either anti-HLA-ABC (W6/32; RPE (C#R7000)) or IGg2a-(RPE; C#X0950) as isotype control. Cells were incubated under low light illumination for 30 min then washed and sorted using a BD FACS Aria III flow cytometer. Cells expressing HLA-ABC were captured and seeded individually into single cells in 96 well tissue culture plates, for clonal expansion.

### Human RT2-Profiler Type 1 IFN PCR arrays and RT-PCR studies

RNA was extracted from cells using an RNeasy Mini kit (Qiagen, Maryland, USA) and its quantity and quality were estimated by NanoDrop measurement (Thermofisher, USA). 500 ng RNA was used for cDNA synthesis (Qiagen) and gene expression monitored by Real-time PCR with SYBR Green master mix using commercially available RT2 Profiler PCR Array and primers for genes of interest (Qiagen, USA). Amplicons were generated on the QuantStudio Flex 12K (Applied Biosystems, California, USA) and gene expression calculated using the comparative threshold cycle method (2^−ΔΔCt^) after normalising with transcripts encoding *HPRT1* and *YY1*.

For RT-PCR experiments, purified RNA was reverse transcribed into cDNA using SuperScript VILO cDNA synthesis Kit (Roche), according to the manufacturer’s instructions. DNA was amplified by cycles of denaturation (95 °C, 30 s) annealing (primer-specific temperature; 30 s) and extension (72 °C; 30 s) in DreamTaq Green PCR Mix (ThermoFisher) for 35–40 cycles. Final extension was for 5 min at 72 °C. Amplified PCR products were separated and visualised after electrophoresis in 1% agarose gels containing 0.005% GelRed. Vomeronasal receptor primer details are provided in Supplementary Table 1.

### Luciferase reporter assays

Cells were transfected for 4–5 h with a luciferase reporter construct under the control of a gamma activated sequence (GAS) promoter using Lipofectamine (7 µL/ug plasmid). Following transfection, human or rat interferon-gamma (20 ng/mL) was added to the cells for 24 h. Cells were lysed and stored at − 20 °C prior to assay according to the manufacturer’s instructions.

### Western blotting

Cell lysates were prepared in 25% LDS buffer (Fisher) containing 10% β-mercaptoethanol and 30 µg protein from each sample loaded on 4–12% pre-cast gels (Invitrogen) prior to electrophoresis. Proteins were transferred to PVDF membranes then incubated with primary antibodies overnight at 4 °C. Membranes were washed and probed with alkaline phosphatase tagged secondary antibodies for 1 h at room temperature. Labelling was detected using CDP-star reagent and a Licor C-Digit scanner. GAPDH was used as loading control.

### Whole cell patch clamping

Whole cell patch clamp recordings were obtained using a MutliClamp 700A amplifier (Molecular Devices, https://www.moleculardevices.com) and a NIDAQ 6363 (National Instruments, https://www.ni.com/en-gb.html). Stimulation and recording protocols were performed with the Matlab-based Symphony software (OpenEphys, https://open-ephys.org/symphony). Data were analysed with bespoke python code (Anaconda, python 3.6.3, https://anaconda.org).

The extracellular solution contained 2.5 mM glucose, 140 mM NaCl, 5 mM KCl, 2.6 mM CaCl_2_, 1.2 mM MgCl_2_ 10 mM HEPES (pH 7.4 adjusted with NaOH, osmolarity 295 mOsm/L adjusted with sucrose). The intracellular solution was 120 mM KCl, 1 mM CaCl_2_, 1 mM MgCl_2_, 10 mM EGTA, 10 mM HEPES (pH 7.2 adjusted with KOH, osmolarity 285 mOsm/L adjusted with sucrose)^39^.

Cells were plated on 13 mm cover slips and patched at a holding potential of − 60 mV. Voltage steps were applied from − 100 to + 30 mV in 10 mV steps, each lasting 500 ms, with 100 ms between. The sampling rate was set to 20 kHz and filtered using a 4-pole Bessel filter at 4 kHz. Currents were recorded from individual cells and scaled by their estimated whole cell capacitance following whole cell compensation.

### Human growth hormone secretion assays

Confluent 1.1B4 cells were transiently transfected using lipofectamine for 24 h (37 °C) with 0.5 µg of a plasmid encoding human growth hormone (hGH) kindly provided by Prof Guy Rutter (Imperial College, London). They were washed and the culture medium replaced for a further 24 h. They were then incubated in pre-warmed Krebs Ringer Buffer (KRB) (125 mM NaCl, 4.74 mM KCl, 1 mM CaCl_2_, 1.2 mM KH_2_PO_4_, 1.2 mM MgSO_4_, 5 mM NaHCO_2_, 25 mM HEPES) containing 0.1% BSA for 1.5 h prior to addition of relevant test reagents. Following incubation, supernatants were collected and stored at − 20 °C prior to assay by ELISA.

### Human growth hormone ELISA

hGH secretion was assessed by ELISA (Roche Cat#11585878001) according to the manufacturer’s instructions and hGH secretion evaluated by reference to a calibration curve constructed in parallel.

### Statistics

The statistical significance of experimental data was assessed by multiple comparison ANOVA with Dunnett’s multiple comparisons test, or Tukey’s post hoc test and data were considered significant when *P* < 0.05.

## Supplementary Information

Supplementary Information.

## Data Availability

The research data supporting this publication are provided within this paper.
