# Supplementary material for: Investigation of the utility of the 1.1B4 cell as a model human beta cell line for study of persistent enteroviral infection
Source: Sci Rep. 2021 Aug 2;11:15624. doi: 10.1038/s41598-021-94878-y (PMC8329048; doi:10.1038/s41598-021-94878-y)
Supplement: Supplementary file 1 — Supplementary Information. [file 41598_2021_94878_MOESM1_ESM.pptx]

## Slide 1
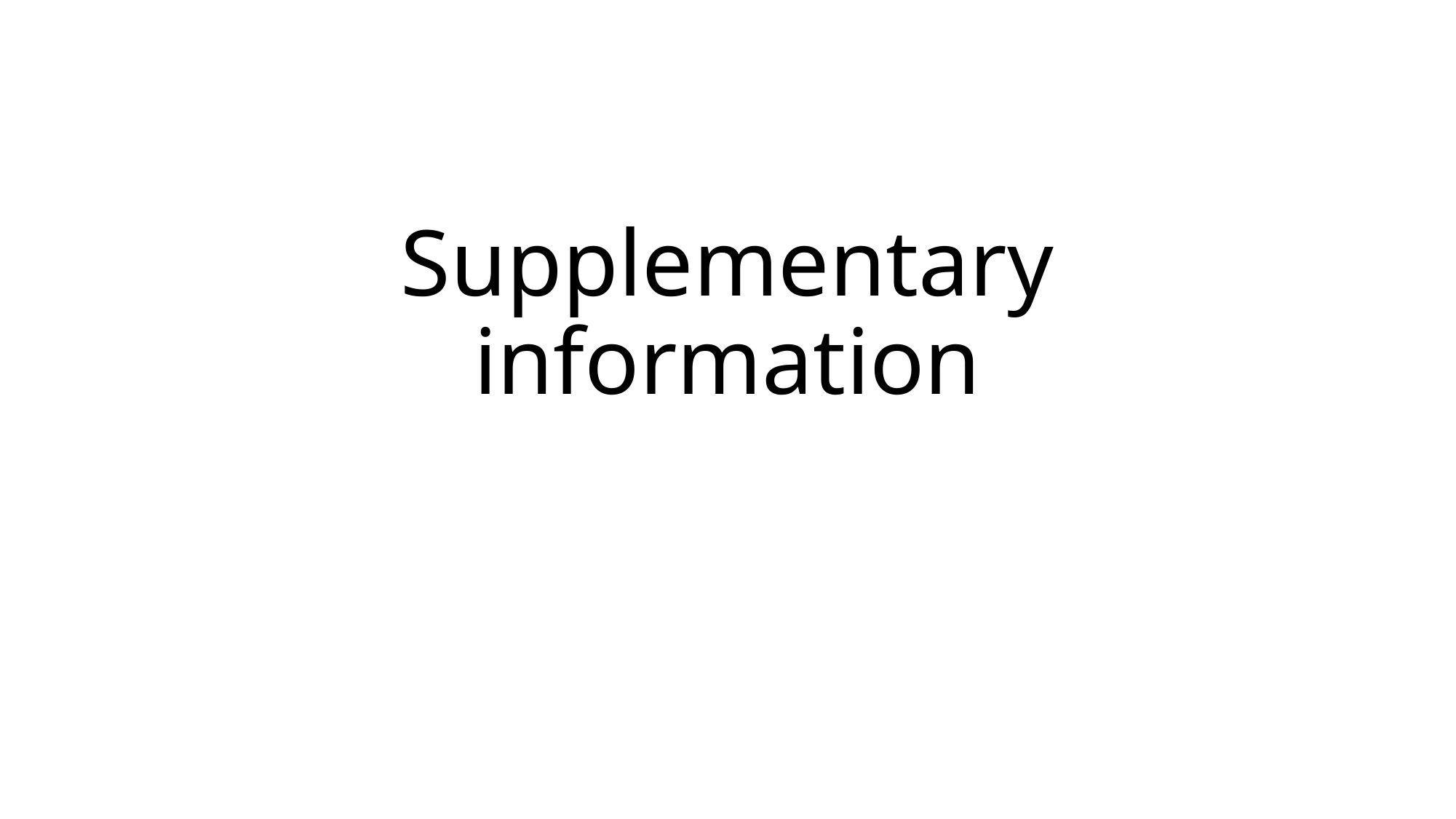

# Supplementary information

## Slide 2
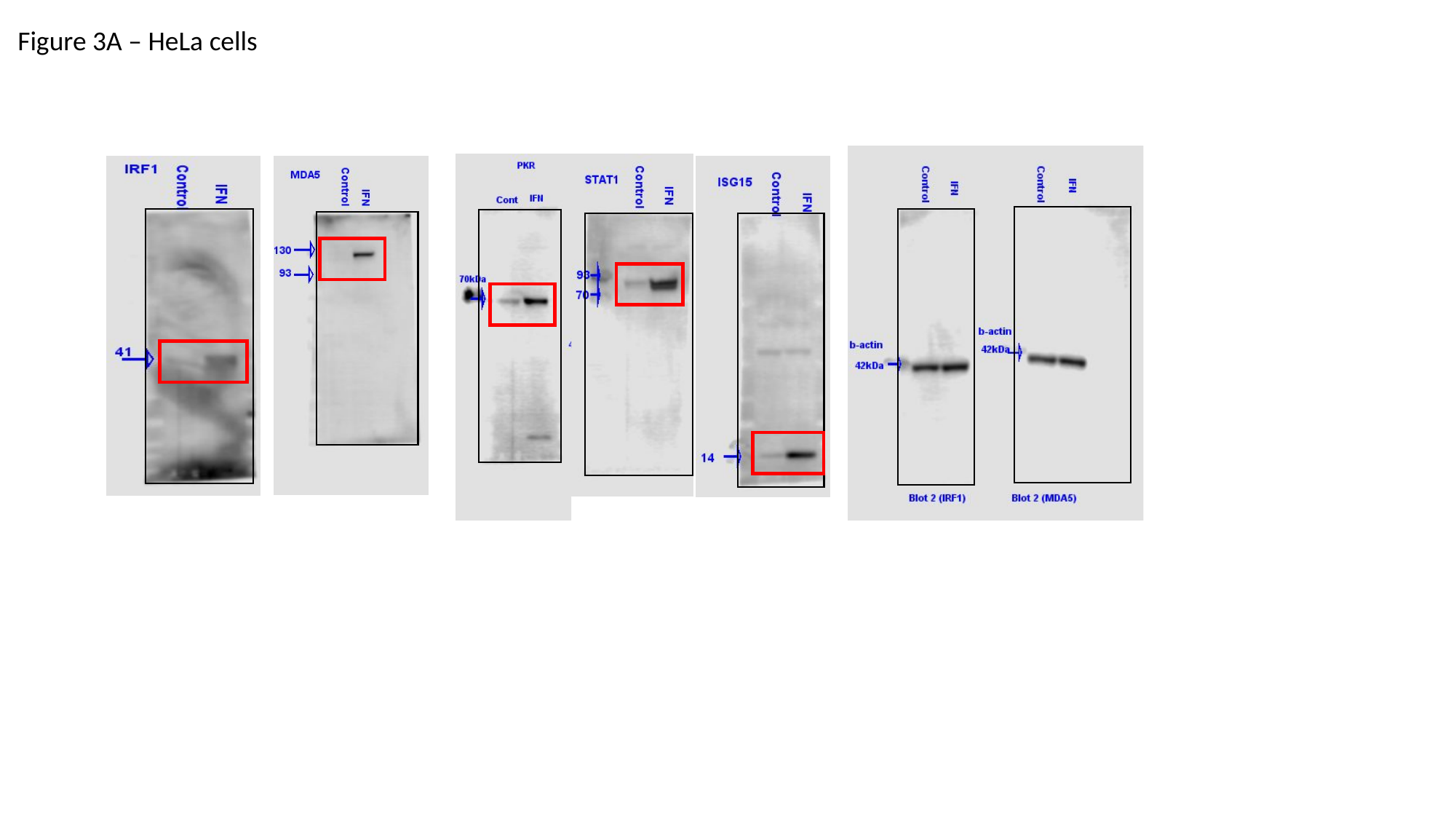

Figure 3A – HeLa cells

## Slide 3
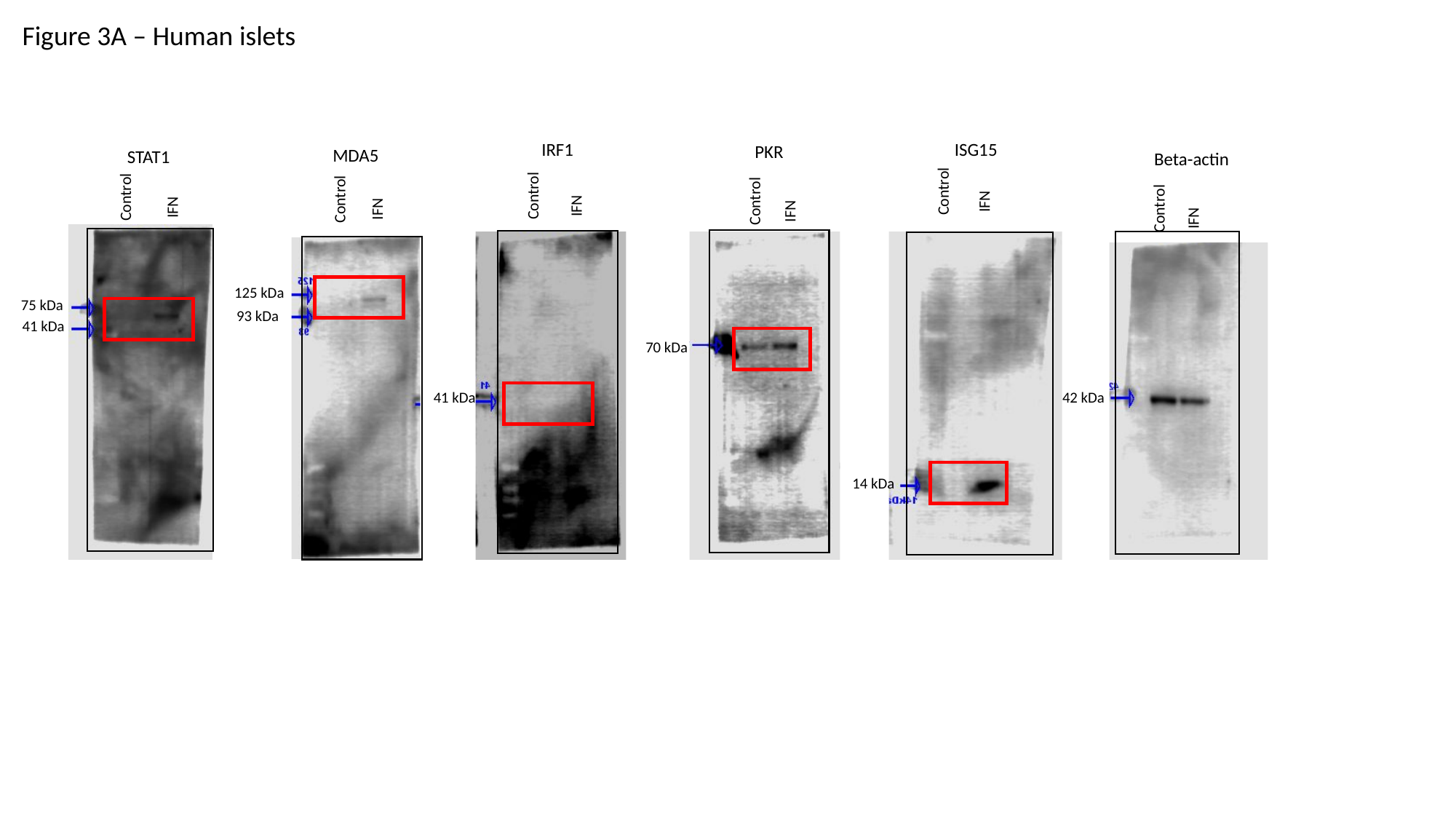

Figure 3A – Human islets
IRF1
Control
IFN
41 kDa
ISG15
Control
IFN
14 kDa
PKR
Control
IFN
70 kDa
MDA5
Control
IFN
125 kDa
93 kDa
STAT1
Control
IFN
75 kDa
41 kDa
Beta-actin
Control
IFN
42 kDa

## Slide 4
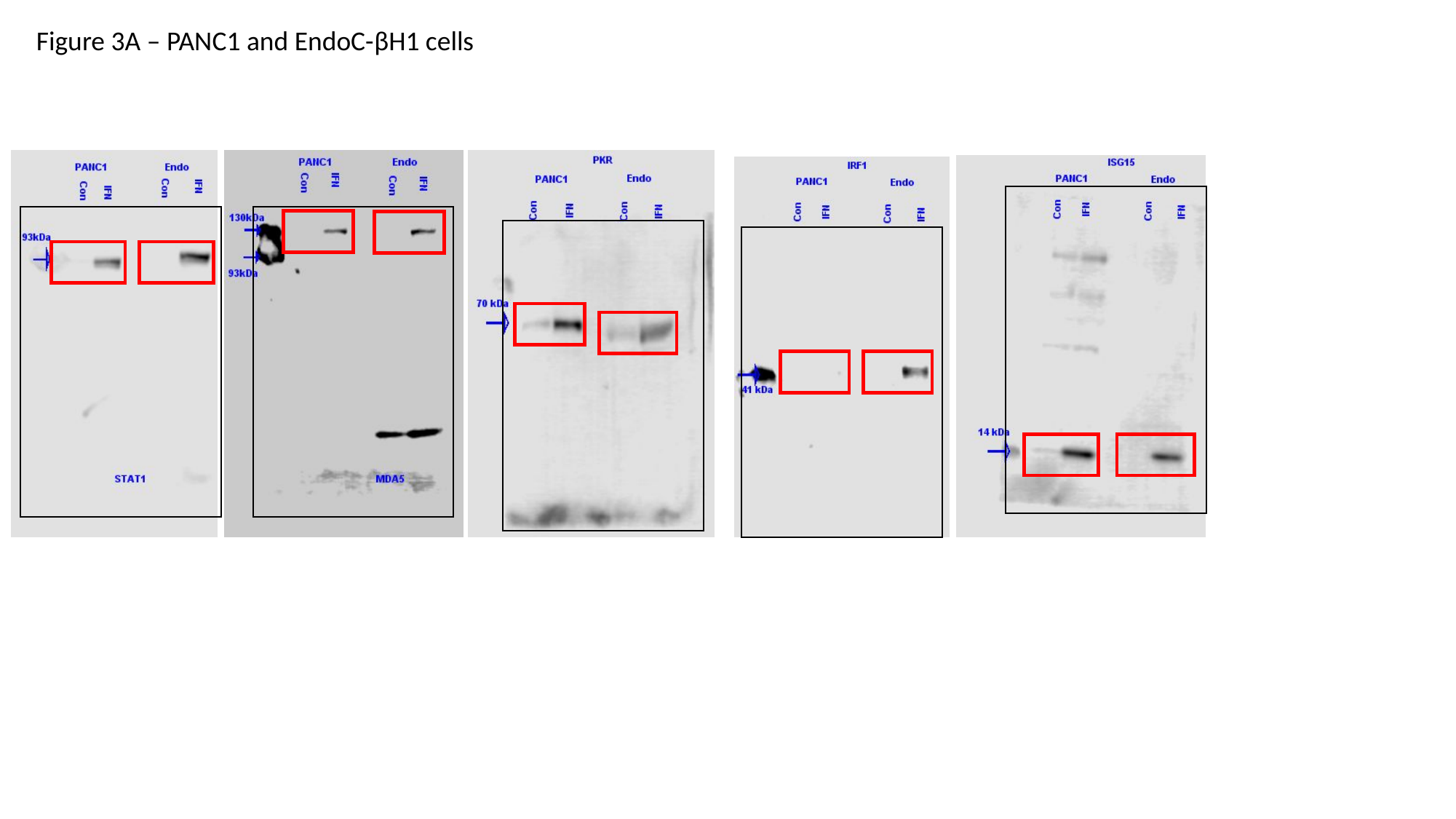

Figure 3A – PANC1 and EndoC-βH1 cells

## Slide 5
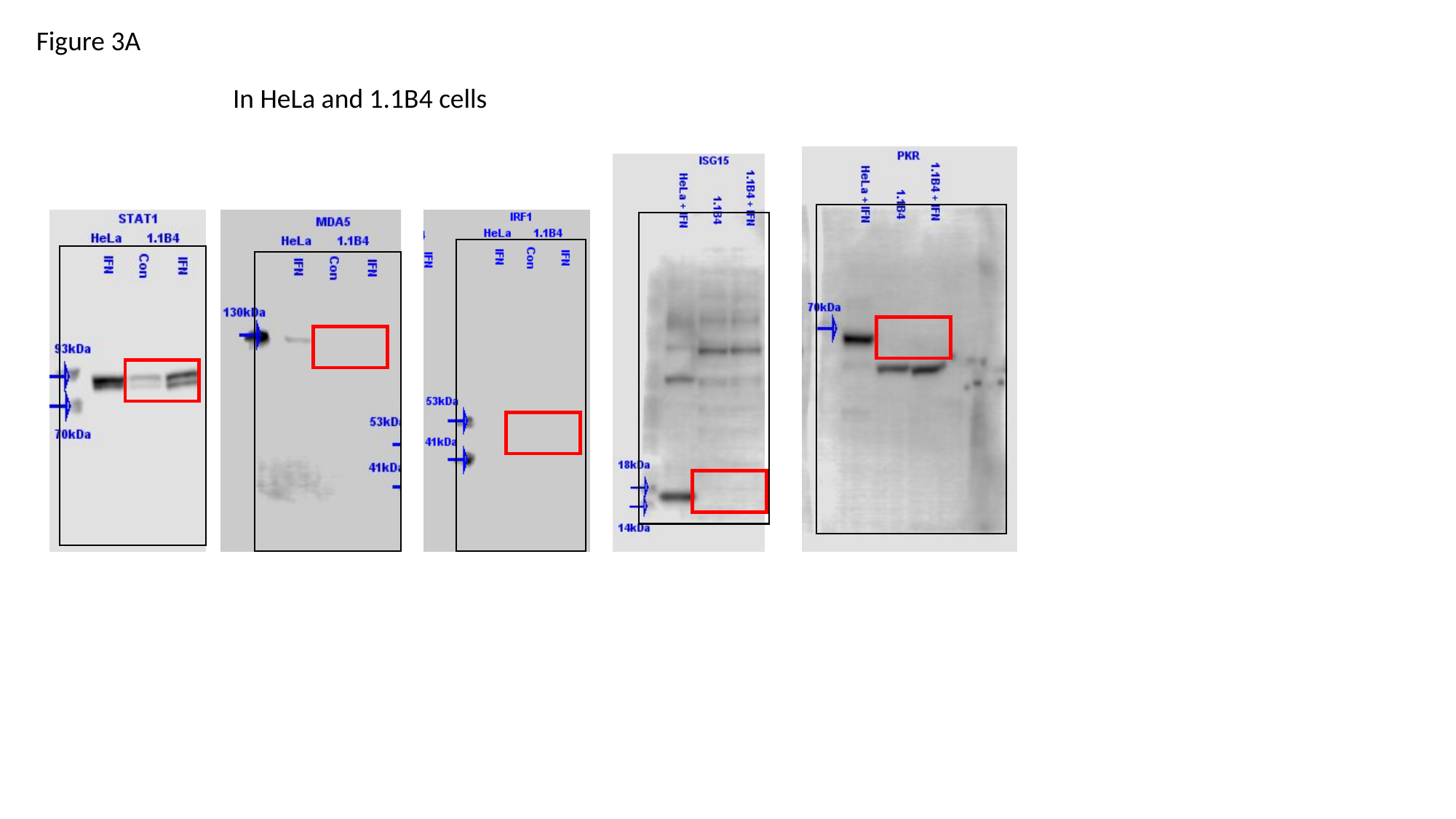

Figure 3A
In HeLa and 1.1B4 cells

## Slide 6
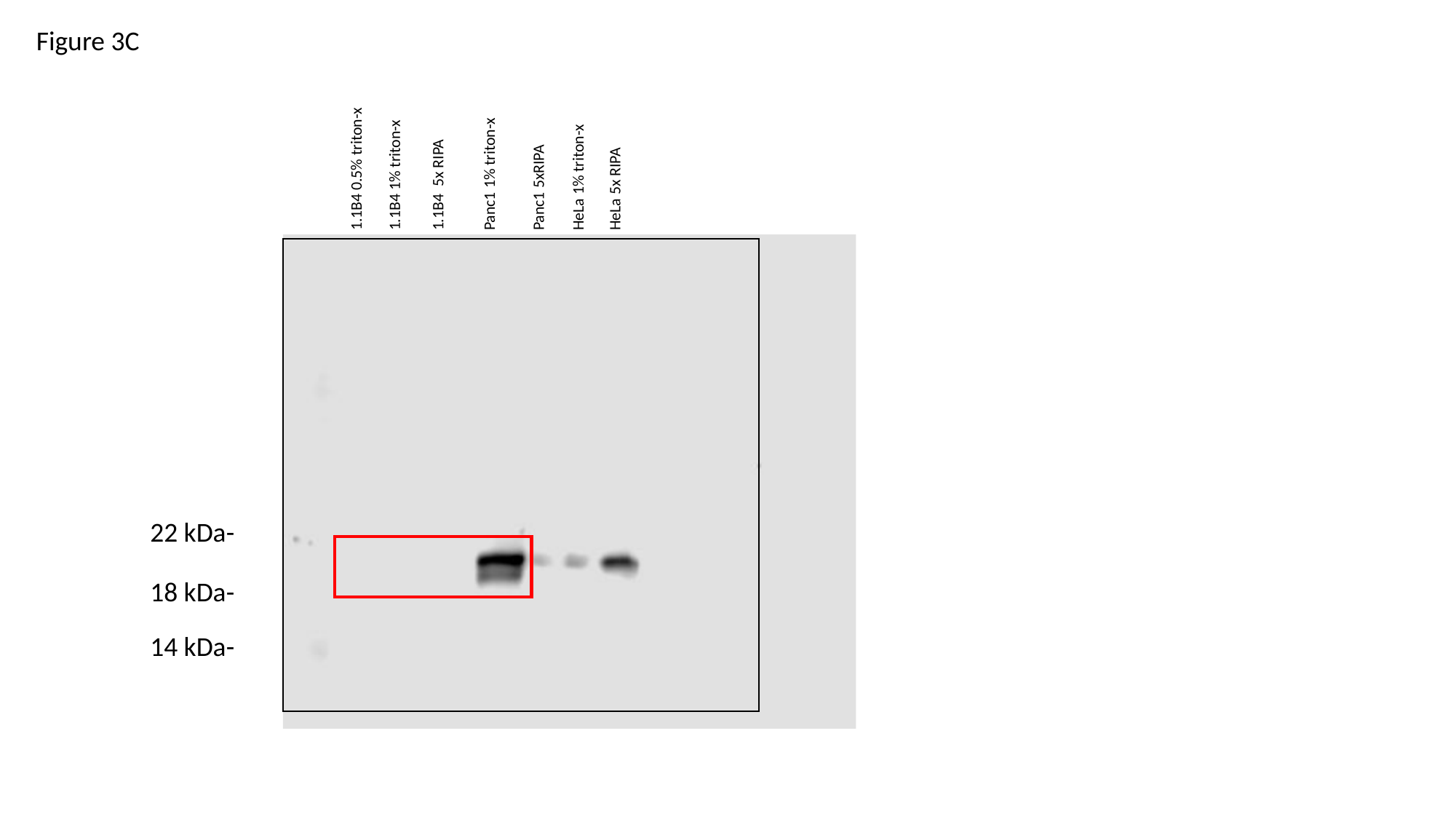

Figure 3C
1.1B4 0.5% triton-x
1.1B4 1% triton-x
1.1B4 5x RIPA
Panc1 1% triton-x
Panc1 5xRIPA
HeLa 1% triton-x
HeLa 5x RIPA
22 kDa-
18 kDa-
14 kDa-

## Slide 7
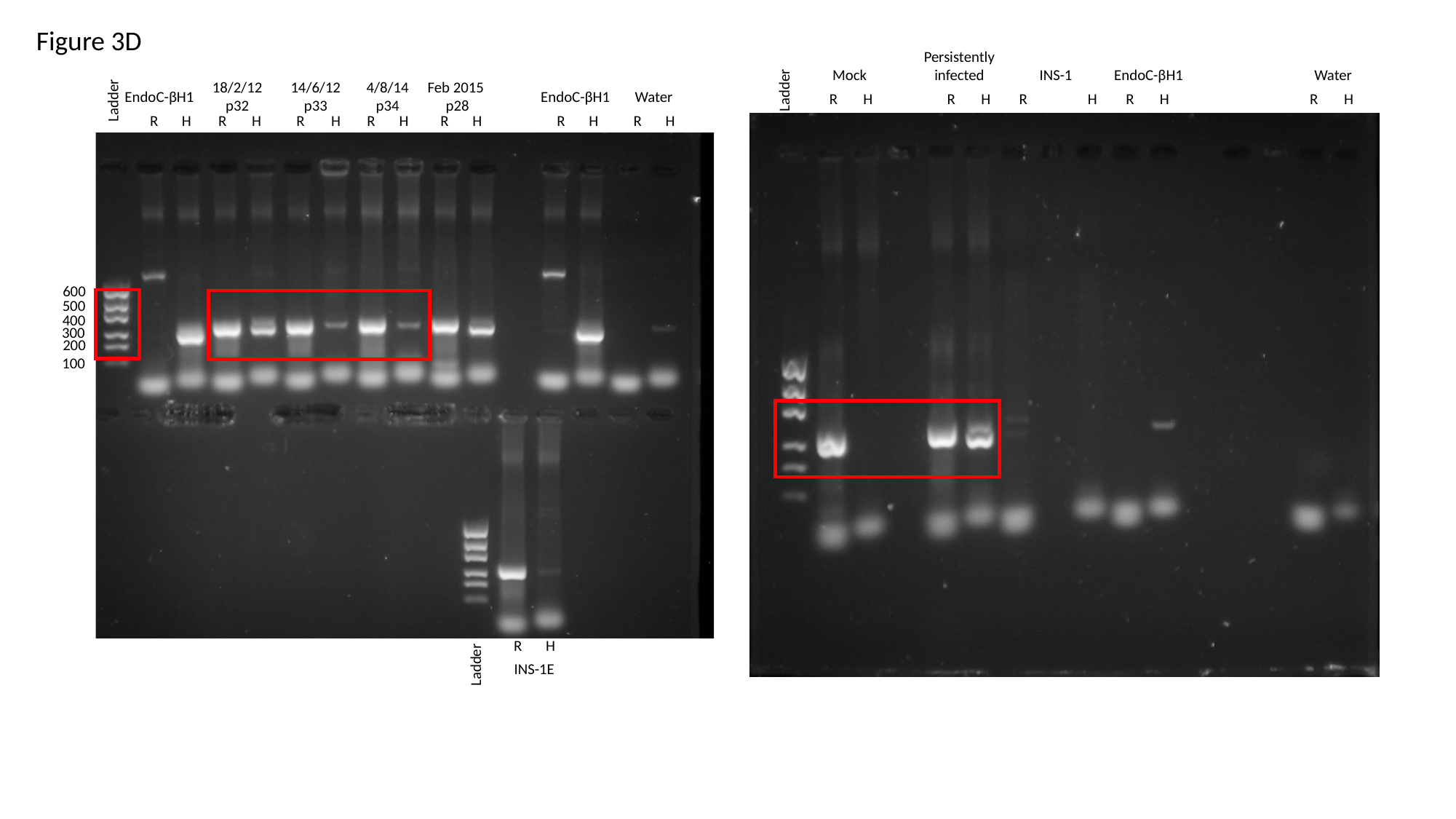

Figure 3D
Persistently infected
Mock
INS-1
EndoC-βH1
Water
18/2/12
p32
14/6/12
p33
4/8/14
p34
Feb 2015
p28
Ladder
EndoC-βH1
EndoC-βH1
Water
R
H
R
H
R
H
R
H
R
H
Ladder
R
H
R
H
R
H
R
H
R
H
R
H
R
H
600
500
400
300
200
100
R
H
Ladder
INS-1E

## Slide 8
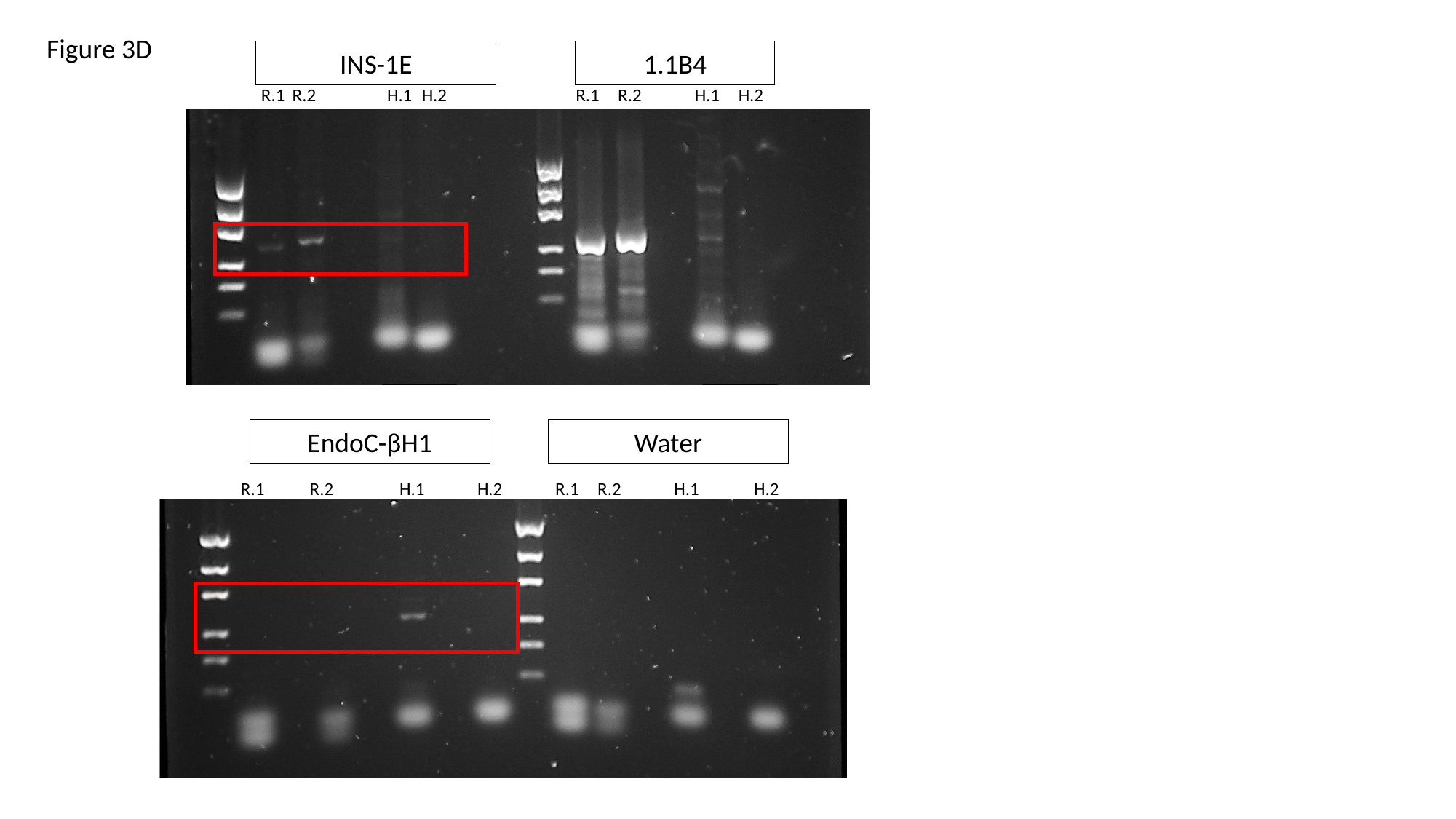

Figure 3D
INS-1E
1.1B4
R.1
R.2
H.1
H.2
R.1
R.2
H.1
H.2
EndoC-βH1
Water
R.1
R.2
H.1
H.2
R.1
R.2
H.1
H.2

## Slide 9
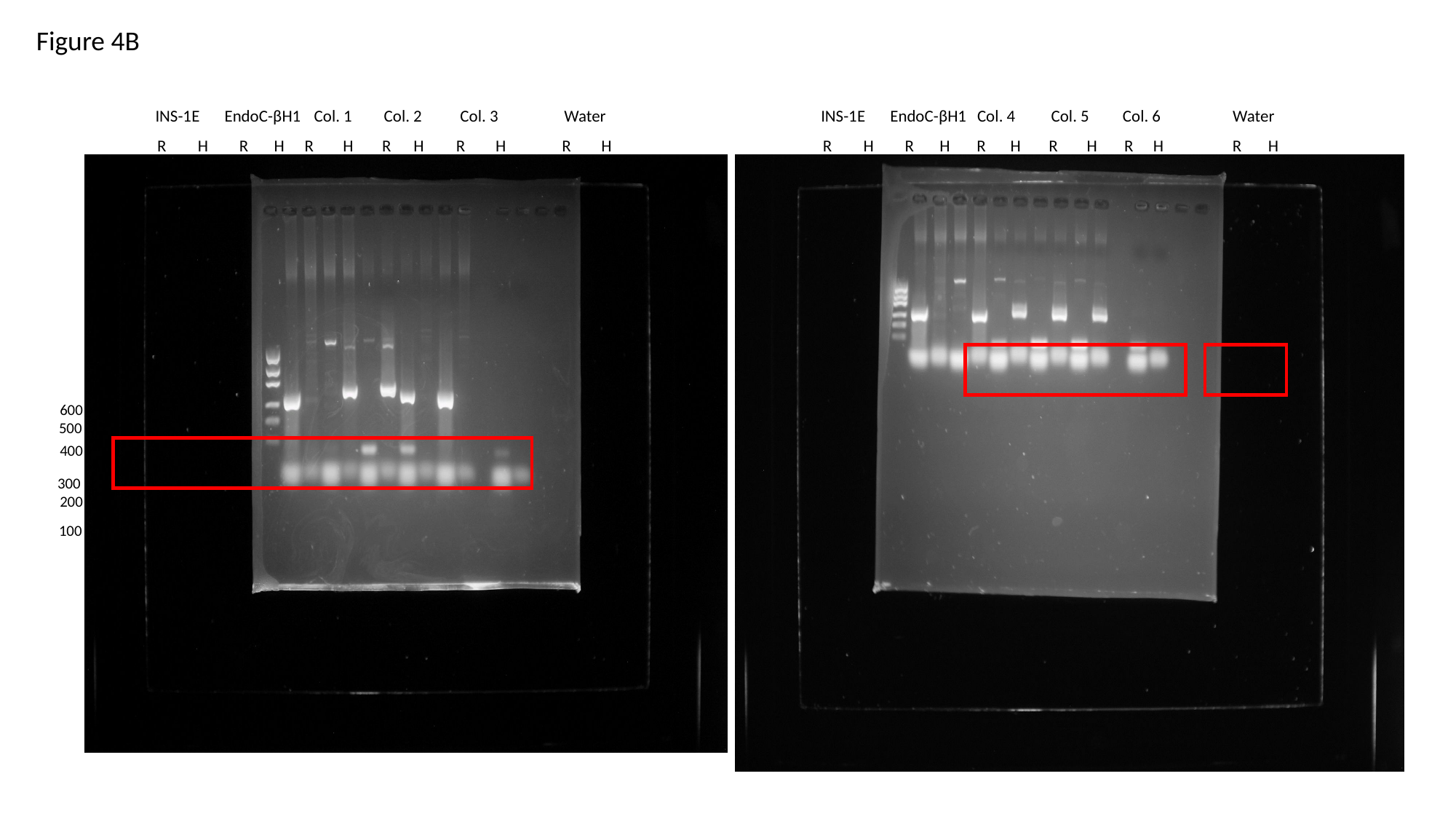

Figure 4B
INS-1E
EndoC-βH1
Col. 1
Col. 2
Col. 3
Water
INS-1E
EndoC-βH1
Col. 4
Col. 5
Col. 6
Water
R
H
R
H
R
H
R
H
R
H
R
H
R
H
R
H
R
H
R
H
R
H
R
H
600
500
400
300
200
100

## Slide 10
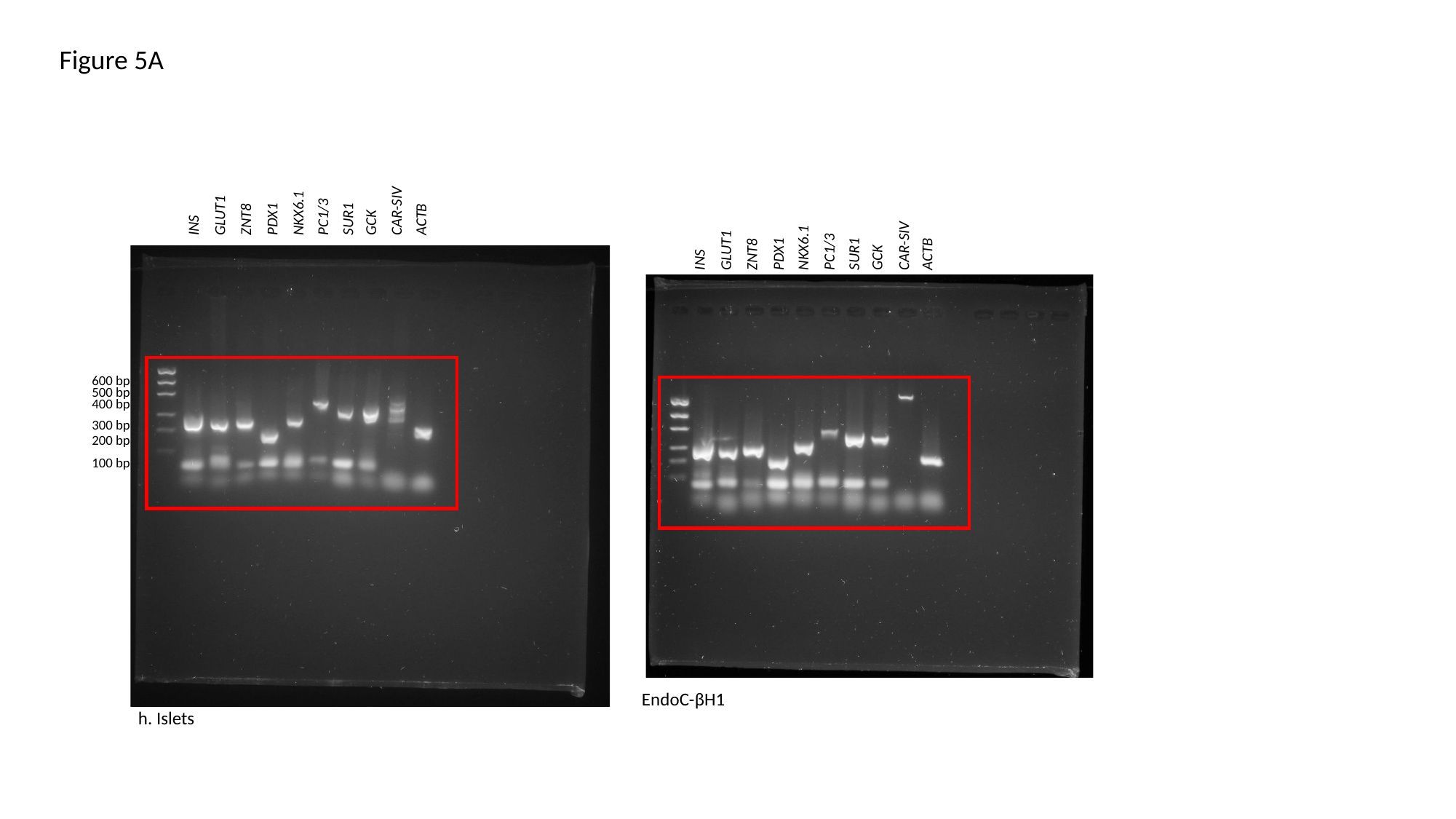

Figure 5A
NKX6.1
CAR-SIV
GLUT1
PC1/3
SUR1
GCK
ACTB
ZNT8
PDX1
INS
NKX6.1
CAR-SIV
GLUT1
PC1/3
SUR1
GCK
ACTB
ZNT8
PDX1
INS
600 bp
500 bp
400 bp
300 bp
200 bp
100 bp
EndoC-βH1
h. Islets

## Slide 11
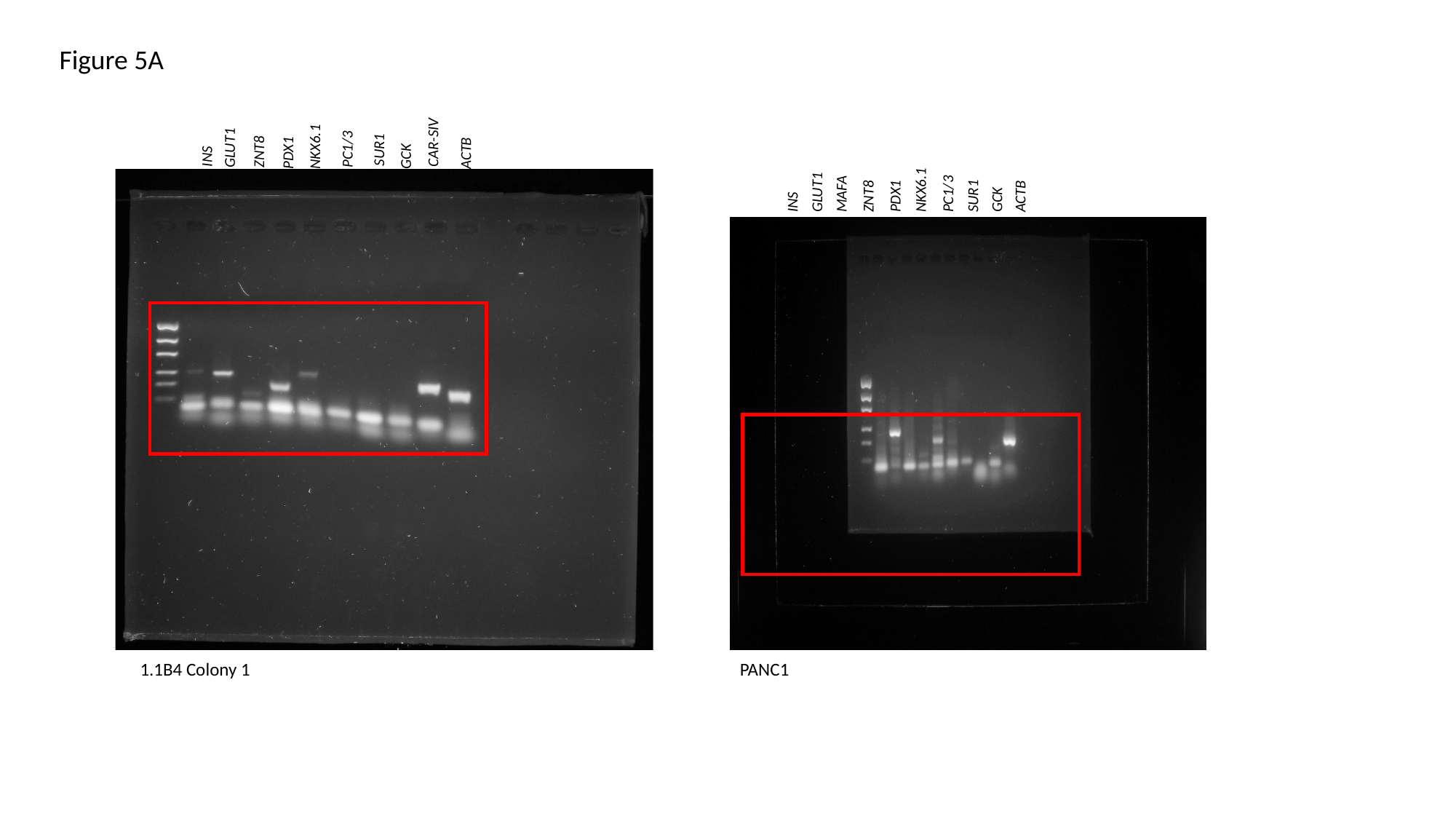

Figure 5A
CAR-SIV
NKX6.1
SUR1
GLUT1
PC1/3
GCK
ACTB
ZNT8
PDX1
INS
NKX6.1
GLUT1
MAFA
PC1/3
SUR1
GCK
ACTB
ZNT8
PDX1
INS
PANC1
1.1B4 Colony 1

## Slide 12
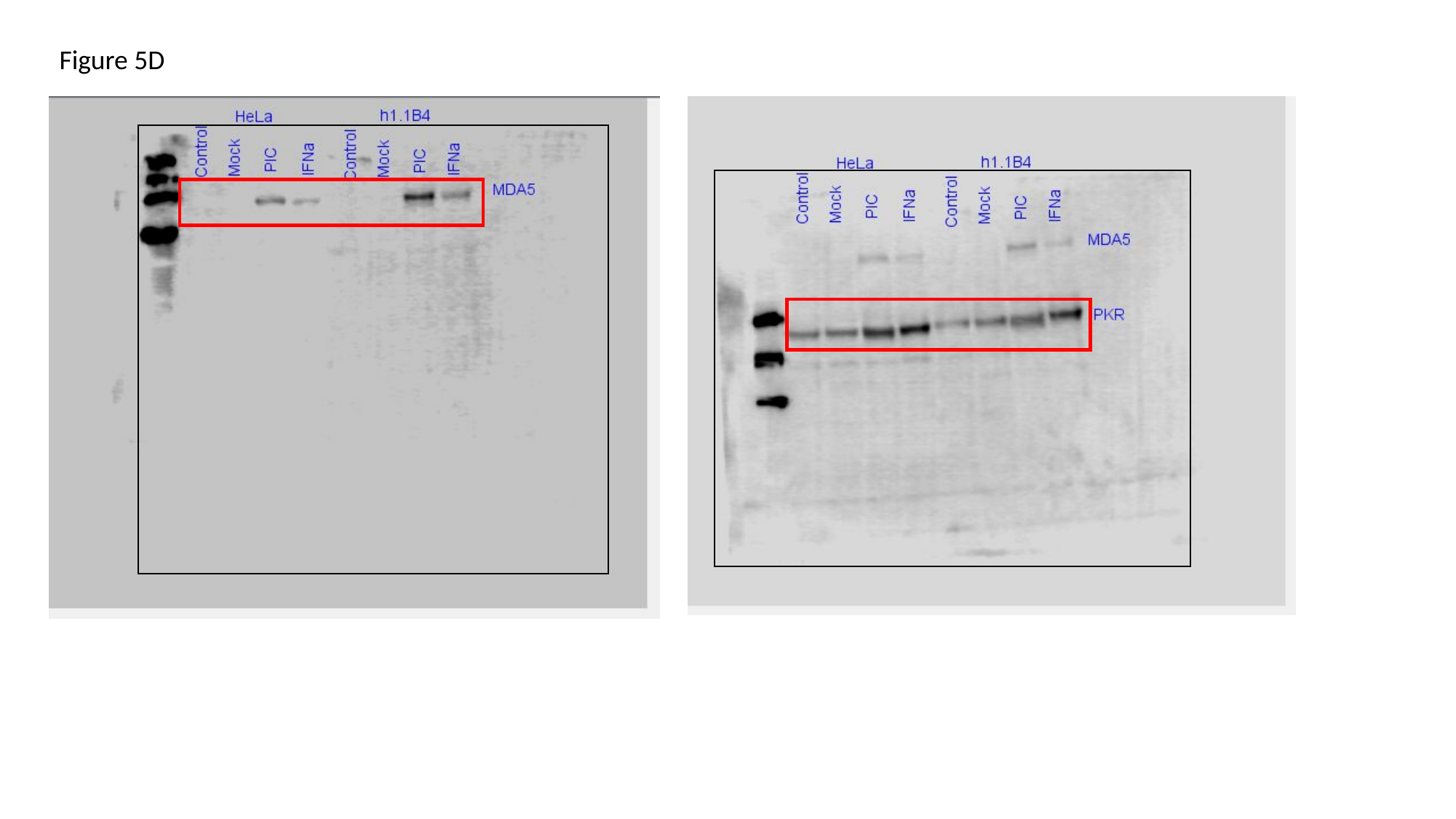

Figure 5D

## Slide 13
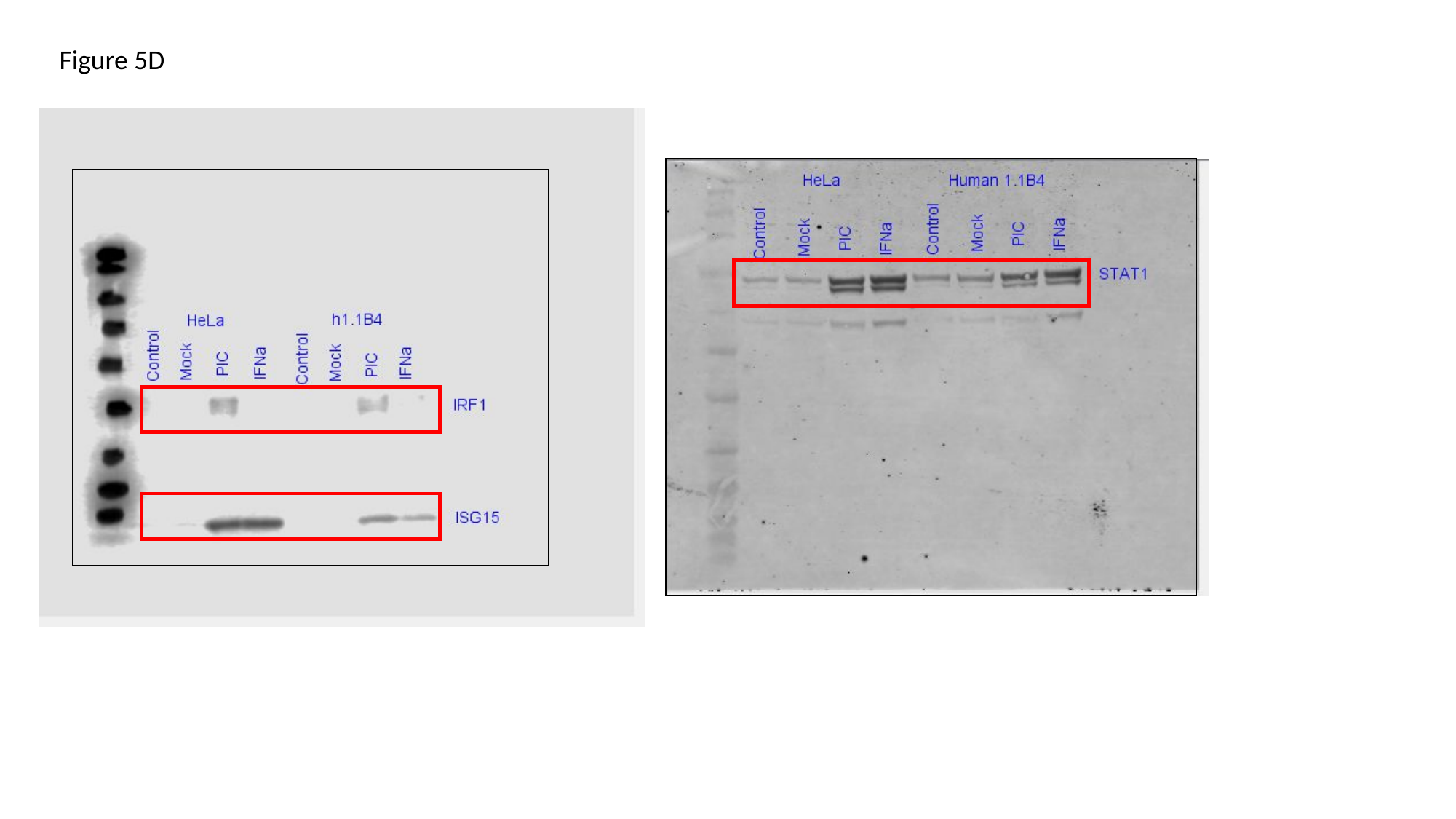

Figure 5D

## Slide 14
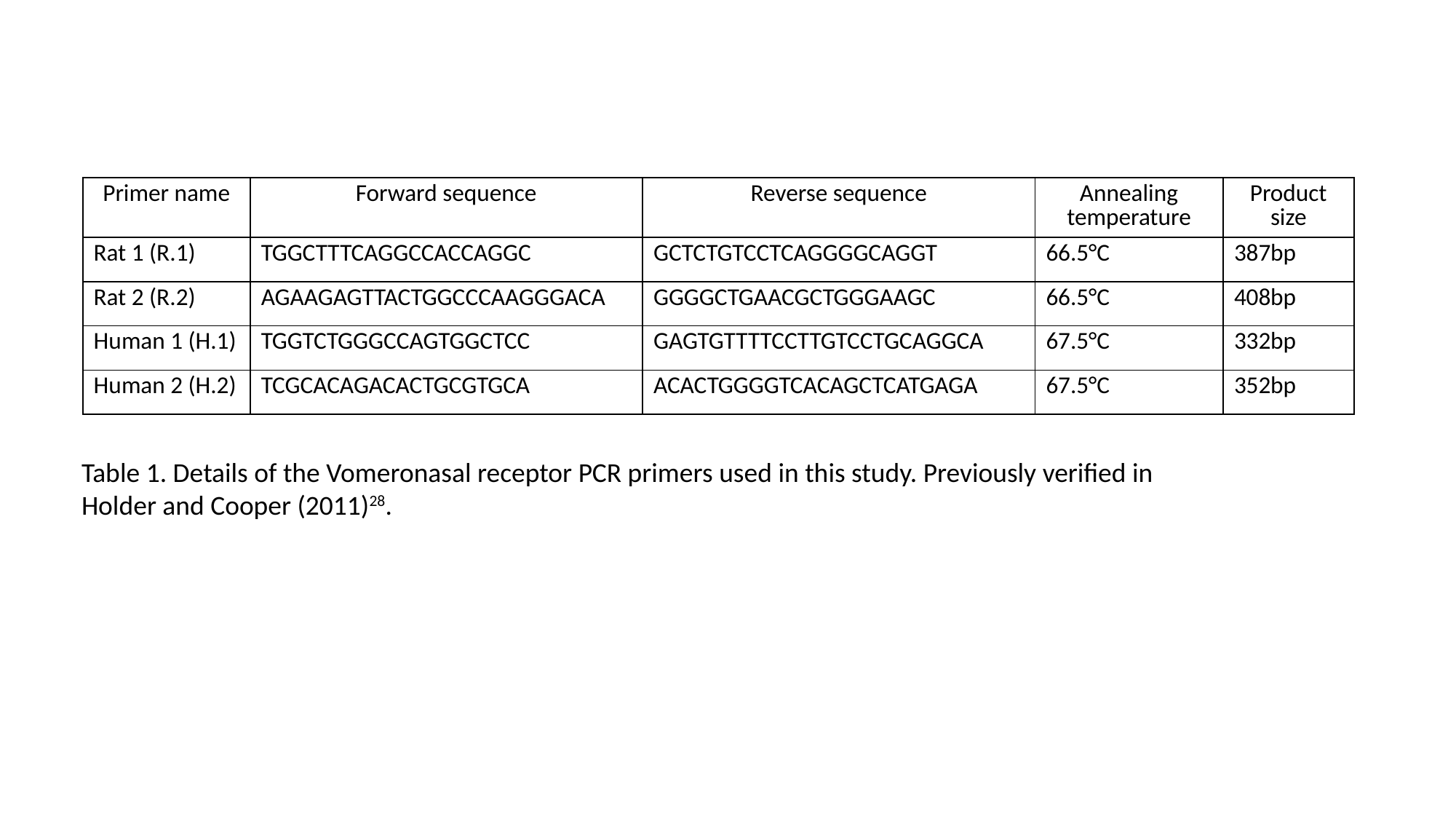

| Primer name | Forward sequence | Reverse sequence | Annealing temperature | Product size |
| --- | --- | --- | --- | --- |
| Rat 1 (R.1) | TGGCTTTCAGGCCACCAGGC | GCTCTGTCCTCAGGGGCAGGT | 66.5°C | 387bp |
| Rat 2 (R.2) | AGAAGAGTTACTGGCCCAAGGGACA | GGGGCTGAACGCTGGGAAGC | 66.5°C | 408bp |
| Human 1 (H.1) | TGGTCTGGGCCAGTGGCTCC | GAGTGTTTTCCTTGTCCTGCAGGCA | 67.5°C | 332bp |
| Human 2 (H.2) | TCGCACAGACACTGCGTGCA | ACACTGGGGTCACAGCTCATGAGA | 67.5°C | 352bp |
Table 1. Details of the Vomeronasal receptor PCR primers used in this study. Previously verified in Holder and Cooper (2011)28.
